# Transcriptome analysis of different growth stages of *Aspergillus oryzae* reveals dynamic changes of distinct classes of genes during growth

**DOI:** 10.1186/s12866-018-1158-z

**Published:** 2018-02-14

**Authors:** Bin He, Zhihong Hu, Long Ma, Haoran Li, Mingqiang Ai, Jizhong Han, Bin Zeng

**Affiliations:** Jiangxi Key Laboratory of Bioprocess Engineering and Co-Innovation Center for In-vitro Diagnostic Reagents and Devices of Jiangxi Province, College of Life Sciences, Jiangxi Science & Technology Normal University, Nanchang, 330013 China

**Keywords:** *Aspergillus oryzae*, Transcriptome, Growth stages, Gene clusters

## Abstract

**Background:**

The gene expression profile and metabolic pathways of *Aspergillus oryzae* underlying the anatomical and morphological differentiation across different growth stages have not been fully characterized. The rapid development of next-generation sequencing technologies provides advanced knowledge of the genomic organization of *A. oryzae*.

**Results:**

In this study, we characterized the growth and development of *A. oryzae* at different growth stages, including the adaptive phase, logarithmic phase, and stationary phase. Our results revealed that *A. oryzae* undergoes physiological and morphological differentiation across the different stages. RNA-seq was employed to analyze the three stages of *A. oryzae*, which generated more than 27 million high-quality reads per sample. The analysis of differential gene expression showed more genes expressed differentially upon transition from the adaptive phase to the logarithmic and stationary phases, while relatively steady trend was observed during the transition from the logarithmic phase to the stationary phase. GO classification of the differentially expressed genes among different growth stages revealed that most of these genes were enriched for single-organism process, metabolic process, and catalytic activity. These genes were then subjected to a clustering analysis. The results showed that the cluster with the majority of genes with increased expression upon transition from the adaptive phase to the logarithmic phase, and steady expression from the logarithmic phase to the stationary phase was mainly involved in the carbohydrate and amino acid metabolism.

**Conclusion:**

Our results provide a foundation for identifying developmentally important genes and understanding the biological processes across various growth stages.

**Electronic supplementary material:**

The online version of this article (10.1186/s12866-018-1158-z) contains supplementary material, which is available to authorized users.

## Background

*Aspergillus oryzae* (*A. oryzae*) has been widely used for the large scale production of food products such as sake, miso, and soy sauce, for more than ten centuries. In addition, *A. oryzae* has been listed as “Generally Recognized as Safe (GRAS)” by the Food and Drug Administration (FDA) in the USA and its safety was supported by the World Health Organization (WHO) [1]. Owing to the important role of *A. oryzae* in industrial production and the general scientific interest, it has been intensively studied in the past century, with most investigations focusing on the breeding techniques for industrial production and for the molecular elucidation for safety [2, 3]. Tominaga et al. reported that the homologous gene cluster for aflatoxin biosynthesis was not expressed in *A. oryzae* even under conditions that are favorable to aflatoxin expression in *A. flavus* [4]. Several studies have also found that genes encoding hydrolases and transporters were induced in solid-state cultivation, which might suggest that gene regulation of *A. oryzae* may be fine-tuned for degrading raw materials with a high level of safety [5]. Meanwhile, there were more sensor histidine kinases in *A. oryzae* than in *Saccharomyces cerevisiae* (*S. cerevisiae*), whereas the numbers of response regulators and histidine-containing phosphotransfer factors were mostly consistent [6]. These characteristics may help *A. oryzae* acquire the ability to adapt to various growth conditions and applied to solid-state fermentation. *A. oryzae* undergoes anatomical and morphological differentiation across different growth stages, which is an tissue of considerable interest, notably due to the influence of morphology on process productivity [7]. However, the knowledge of the fundamental biological processes of *A. oryzae* across different growth stages including the adaptive phase, logarithmic phase, and stationary phase, especially the gene expression profile and metabolic pathways, is limited when compared to *S. cerevisiae* and some *Aspergillus* fungi. Thus, a more comprehensive analysis of the gene regulation mechanisms in *A. oryzae* development would be helpful to improve the understanding of the possible biological processes and will be beneficial for industrial breeding.

More recently, genome-wide approaches have become a primary strategy in characterizing gene expression profililes and elucidating the genetic networks in all fields, including plants, animals and microorganisms [8–10]. The genome-wide projects of *A. oryzae* involved a lot of effort, and the complete genome sequence of *A. oryzae* was obtained using the whole-genome shotgun approach in 2005, which contained a total of 12,074 genes and comprised of eight chromosomes [11]. Akao et al. analyzed the expressed sequence tags from *A. oryzae* cultured in different conditions, including liquid nutrient-rich culture, liquid maltose-inductive culture, and liquid carbon-starved culture [12]. The transcriptomes of *A. oryzae* grown on solid-state culture, and in liquid culture with and without DTT treatment were also analyzed to understand the complex transcriptome of *A. oryzae* at the whole genome level [13]. Comparative genome analysis between different *A. oryzae* strains was also performed to reveal a close relationship between sites of mutation and regions of highly divergent genes among various *Aspergillus* species [14]. Several studies have focused on the cell biology of *A. oryzae* across different growth and developmental stages [3], but the transcriptional processes among the growth stages still remain unclear. The transcriptional processes during the growth of *A. oryzae* are now amenable after the completion of its whole genome sequence. Our initial studied found that *A. oryzae* undergoes morphological differentiation across the different growth stages. To better understand the transcriptional processes occurring during the growth stages of *A. oryzae*, we analyzed the three growth phases (the adaptive phase, logarithmic phase, and stationary phase) of this organism by RNA-seq. In this study, we provide a comprehensive transcriptome of *A. oryzae* at each stage, which remarkably enlarges the genomic resources of *A. oryzae* available in the public database. Based on the expression patterns, differentially expressed genes between different growth stages and functional categories of each gene clusters were characterized; these are of great importance to improve understanding of the possible biological processes across various growth stages and enhance efforts for the genetic improvement of the *A. oryzae* fermentative strain.

## Results

### Growth and development of *A. oryzae* during different growth stages

**Fig. 1:**
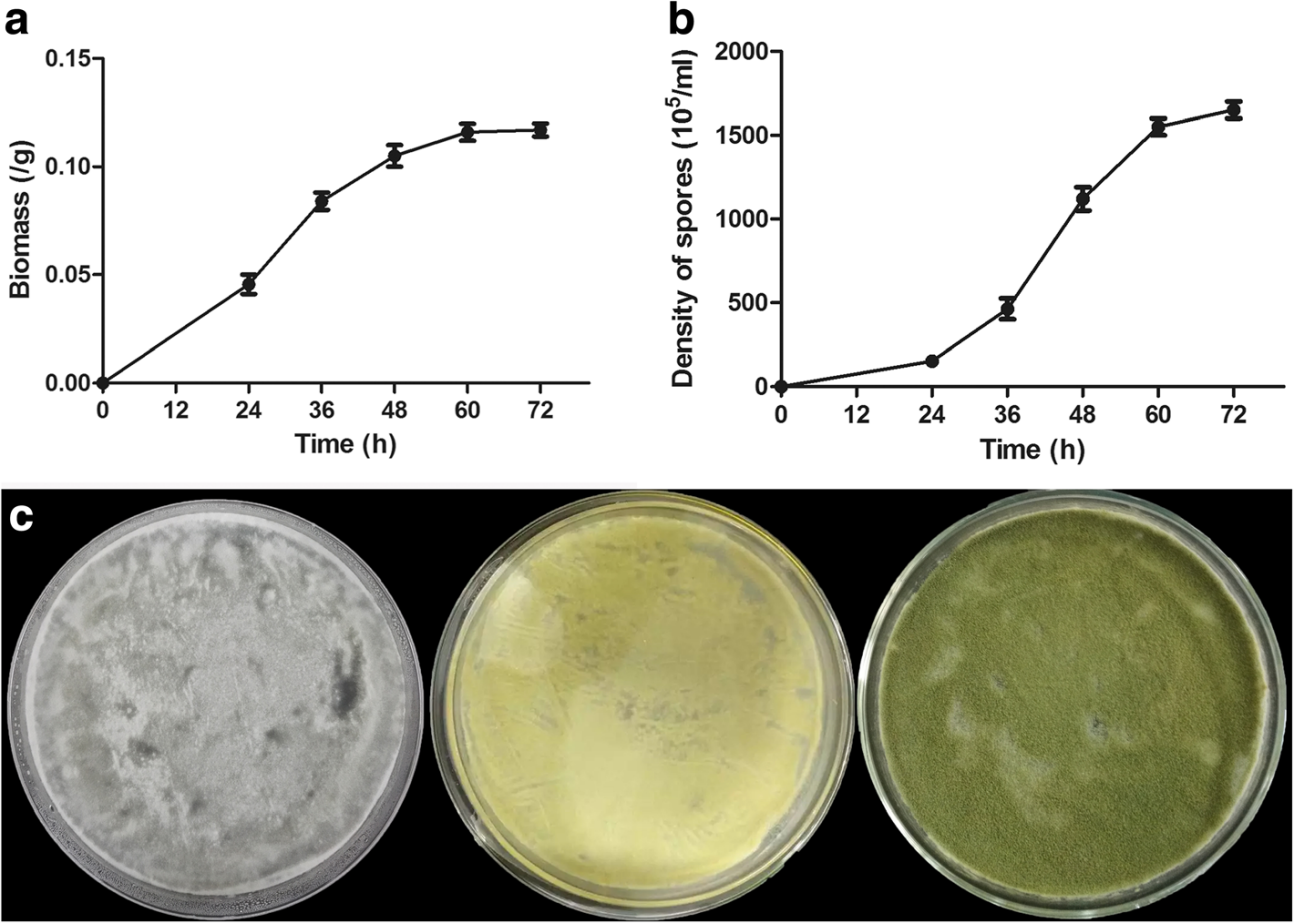
The growth and development of *A. oryzae* at different growth stages. **a** The dry biomass at different growth stages (24 h, 36 h, 48 h, 60 h and 72 h). The mycelia were collected by peeling off from the plates and dried overnight for the determination of biomass; **b** The density of spores in the salinity treatments (24 h, 36 h, 48 h, 60 h and 72 h); **c** The phenotype of *A. oryzae* at different growth stages (from left to right: 24 h, 48 h and 72 h). The color of colony was began as white and then changed from kelly green to olive green

To address the growth and development of *A. oryzae* at various growth stages, a preliminary serial time point study of *A. oryzae* was conducted. Dry biomass and spore density of *A. oryzae* incubated on solid medium were measured to plot the growth curve of *A. oryzae*. During the first 24 h, cells proliferated slowly, and the colonies were white in color. A significant increase in dry biomass and spore density was observed from 24 h to 60 h (Fig. 1). During this time, the colonies turned kelly green in color. After 60 h of incubation, the dry biomass and spore density of *A. oryzae* were relatively steady, and the color of colonies changed to olive green. These results indicated that *A. oryzae* is in an adaptive phase during the first 24 h of growth with slow-growing and white colonies, in logarithmic phase from 24 h to 60 h, in stationary phase after 60 h, accompanied with changes in cell morphology.

### Global analysis of transcriptome

To characterize the patterns of gene expression during growth, three libraries were constructed using samples from various growth stages of *A. oryzae*, with each sample containing three biological replicates (Ao_24_1, 2, 3: samples at 24 h after incubation; Ao_48_1, 2, 3: samples at 48 h after incubation; Ao_72_1, 2, 3: samples at 72 h after incubation). After removing low quality reads and adaptor sequences, more than 27 million high-quality reads per sample were generated for quantification of unigenes (Table 1). The clean reads were aligned to the whole reference genome sequence, and more than 89% of the clean reads for each sample were mapped to the genome (Table 1). The Pearson’s correlation coefficient of gene expression between replicates for each sample was more than 0.905 (Additional file 1: Figure S1). These results indicated our transcriptome data were suitable for further analysis. From these high-quality clean reads, a total of 11,594, 11,651 and 11,694 genes containing 901, 913 and 916 new genes respectively, were obtained from the three growth stages of *A. oryzae*. These new genes will contribute to the re-annotation of the genome for *A. oryzae*.

**Table 1:** Summary of the sequencing data and gene numbers of *A. oryzae* transcriptome at different growth stages

Samples	Raw reads	Clean reads	Mapping Ratio	Known Gene Number	New Gene Number	All Gene Number
Ao_24_1	32,093,940	31,629,822	90.38%	10,693 (93.82%)	901 (6.18%)	11,594
Ao_24_2	36,370,474	35,810,546	89.44%			
Ao_24_3	33,667,860	33,127,436	90.38%			
Ao_48_1	29,986,270	28,507,514	90.35%	10,738 (94.22%)	913 (5.78%)	11,651
Ao_48_2	31,554,924	29,185,736	90.32%			
Ao_48_3	28,855,116	27,530,354	90.87%			
Ao_72_1	32,594,166	29,066,044	90.10%	10,778 (94.57%)	916 (5.43%)	11,694
Ao_72_2	31,561,132	29,580,878	90.09%			
Ao_72_3	36,077,684	30,828,618	87.84%			

### Alternative splicing analysis

**Fig. 2:**
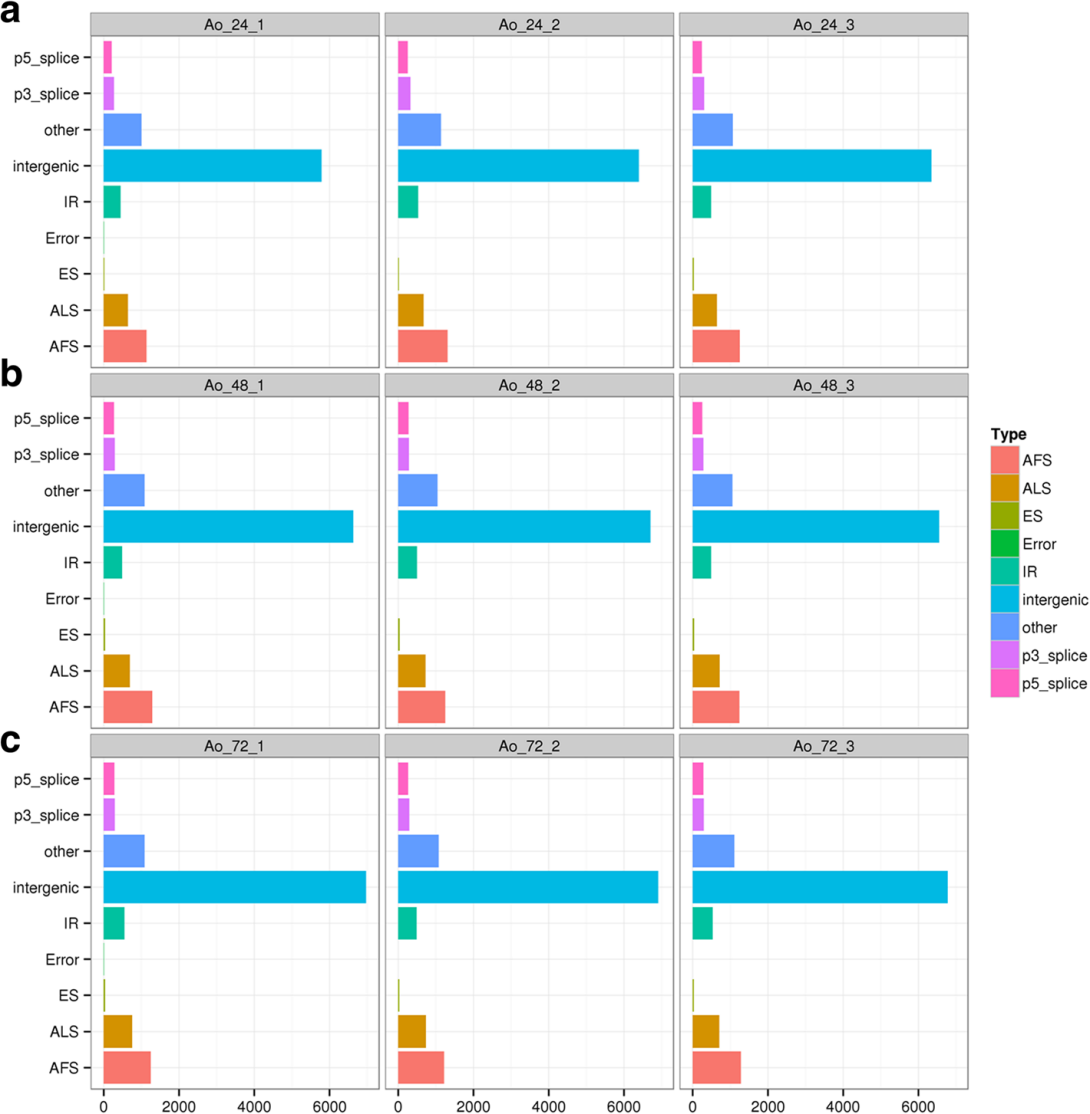
Different types of alternative splicing events at different growth stages. The horizontal axis indicates the type of alternative splicing; the vertical axis indicates the number of alternative splicing. Intergenic (junctions start from and/or end up with the area between genes), P5_splice (junctions start inside an exon, end up with initiation site of another exon), P3_splice (junctions start from an exon termination site and end up inside another exon), ES (junctions start from an exon termination site and end at another termination site), IR (junctions start and end in the same exon), AFS/TSS (junctions start from the 5’ end of exon initiation site and end up with another exon), ALS/TTS (junctions start at the 3’ end of the last exon initiation site and end up with another exon) and Others (junctions that do not belong to any of the above categories). Ao_24_1, 2, 3: samples at 24 h after incubation; Ao_48_1, 2, 3: samples at 48 h after incubation; Ao_72_1, 2, 3: samples at 72 h after incubation

Alternative splicing is pivotal to identify and quantify differentially spliced transcripts for transcriptome analysis [15]. In this study, we classified the alternative splicing sites identified in the nine libraries into eight categories as described in methods. We found that the alternative splicing sites identified among the three biological replicates were highly similar, which confirm the relative consistency of the replicates (Fig. 2). In addition, the alternative splicing events at different growth stages showed a similar trend, and junctions that start at and/or end up in the area between genes were most highly represented, followed by junctions starting at the 5 ‘end of the exon initiation sites and ending with another exon. These results suggested that the number and type of alternative splicing events were not altered during the transition from the adaptive phase to the logarithmic and stationary phase of *A. oryzae*.

### Identification of differentially expressed genes at various growth stages

**Fig. 3:**
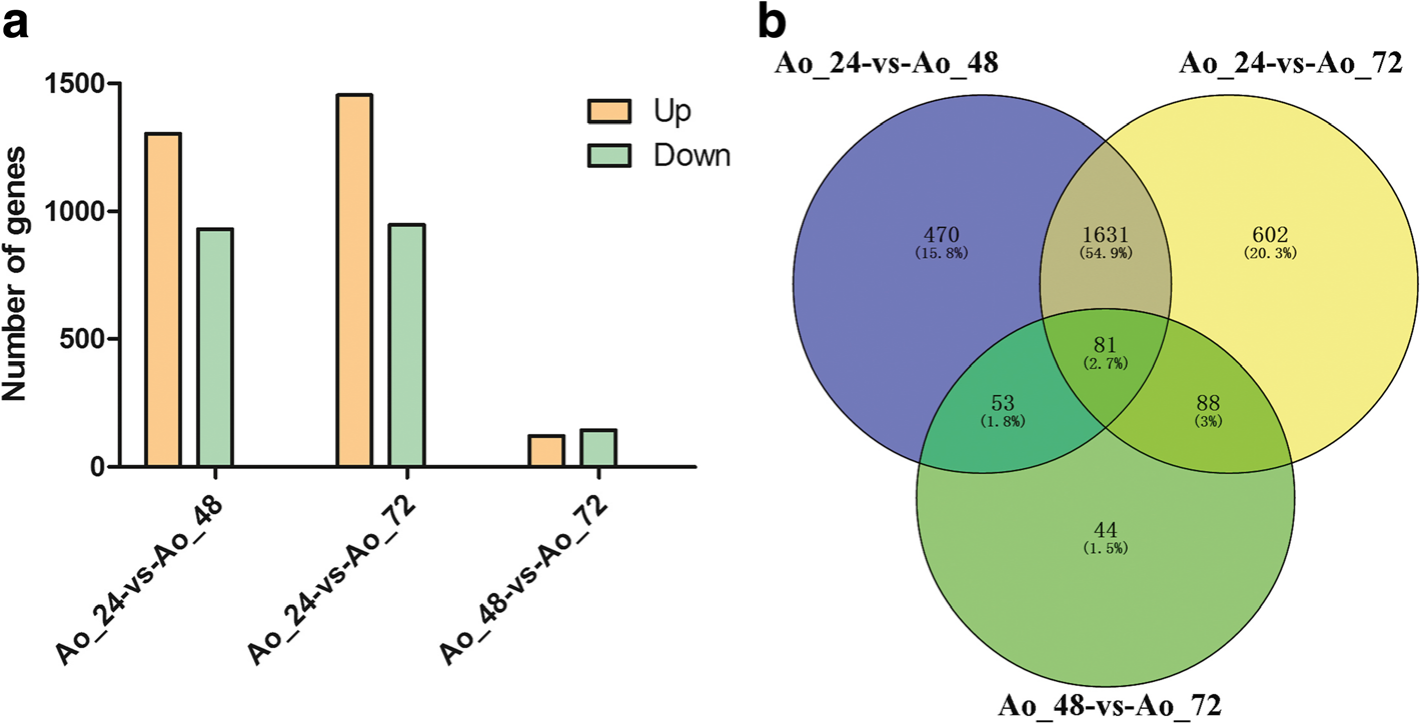
Analysis of DEGs between fungal growth stages. **a** DEGs’ distribution between each two samples; **b** Venn diagram exhibiting the DEUs’ distribution in three libraries. Ao_24: samples at 24 h after incubation; Ao_48: samples at 48 h after incubation; Ao: samples at 72 h after incubation

Differentially expressed genes (DEGs) across different growth stages were identified based on FPKM by applying cutoff of *p*-value < 0.05. A total of 2235 genes were differentially expressed between Ao_24 and Ao_48, and comprised of 1305 up-regulated genes (accounting for 58% of all significant differentially expressed genes) and 930 down-regulated genes (accounting for 42%) (Fig. 3a). There were 2402 significant DEGs between Ao_24 and Ao_72 samples, comprising of 1454 up-regulated genes (61%) and 948 down-regulated genes (39%). Only 266 DEGs were identified between Ao_48 and Ao_72, with 122 up-regulated and 144 down-regulated. A Venn diagram of the distribution of DEGs is shown in Fig. 3b, and we found that 81 DEGs were shared among the three groups. The numbers of specific DEGs in Ao_24-vs-Ao_48 (470, 15.8%) and Ao_24-vs-Ao_72 (602, 20.3%) were remarkably greater than those in Ao_48-vs-Ao_72 (44, 1.5%), indicating the involvement of complex developmental events in the adaptive phase of *A. oryzae* growth. A large proportion of DEGs was found to be common between Ao_24-vs-Ao_48 and Ao_24-vs-Ao_72 and contained 1631 genes, suggesting that they are specifically involved in the developmental processes in the adaptive phase. To further confirm the reliability and availability of DEGs obtained from the RNA-seq analysis, six genes involved in the linoleic acid biosynthesis were selected for qRT-PCR analysis. The results of qRT-PCR were coordinated with those of transcriptome analysis (Additional file 2: Figure S2).

### Functional classification of differentially expressed genes

**Fig. 4:**
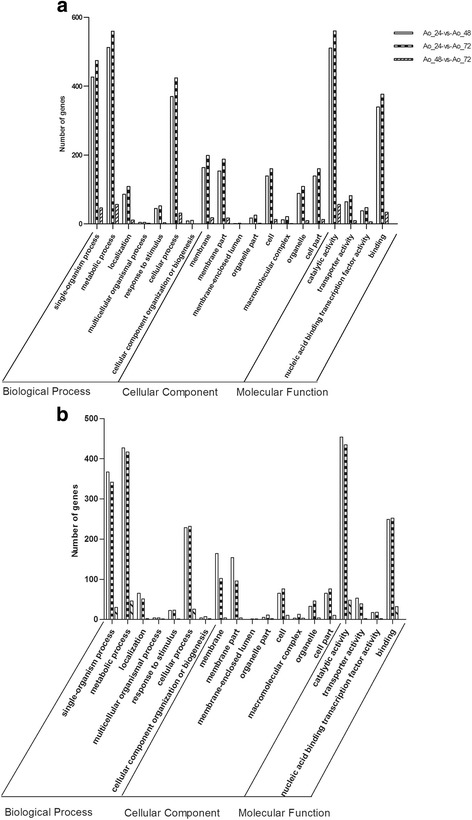
GO enrichment of DEGs between growth stages in *A. oryzae*. **a** The enriched GO terms of up-regulated DEGs between growth stages. **b** The enriched GO terms of down-regulated DEGs between growth stages

To characterize the functional differences between various growth stages of *A. oryzae*, up-regulated and down-regulated DEGs were analyzed initially by GO enrichment to explore the relevant biological functions. Based on the GO terms of transcript, these DEGs were assigned into three main functional categories: cellular component, molecular function and biological process. Apart from unknown categories, oxidation-reduction process (GO: 0055114, subordinated to single-organism process term), generation of precursor metabolites and energy (GO: 0006091, subordinated to metabolic process term) and single-organism cellular process (GO: 0044763, subordinated to cellular process term) were the most highly represented categories between the growth stages. Intrinsic component of membrane (GO: 0031224, subordinated to membrane and cell term) represented the major proportion of the cellular component categories, while oxidoreductase activity (GO: 0016491, subordinated to catalytic activity term) and organic cyclic compound binding (GO: 0097159, subordinated to binding term) were most represented among the various molecular functions. The up-regulated (Fig. 4a) and down-regulated DEGs (Fig. 4b) between different growth stages showed similar GO enrichment patterns. Detailed expression levels and functional annotation of DEGs between Ao_24 and Ao_48, Ao_24 and Ao_72 as well as Ao_48 and Ao_72 are shown in the Additional file 3: Table S2, Additional file 4: Table S3 and Additional file 5: Table S4, respectively.

To further investigate the function of these DEGs, they were mapped to the Kyoto Encyclopedia of Genes and Genomes (KEGG) database and enriched. Between the adaptive phase (Ao_24) and logarithmic phase (Ao_48), degradation of aromatic compounds, tyrosine metabolism and starch and sucrose metabolism comprised the greatest number of DEGs with the minimum Qvalue (Table 2). Between the adaptive phase (Ao_24) and stationary phase (Ao_72), the maps with minimum Qvalue were tyrosine metabolism as well as pentose and glucuronate interconversions, while genes with the minimum Qvalue between the logarithmic phase (Ao_48) and stationary phase (Ao_72) were involved in the carotenoid biosynthesis and terpenoid backbone biosynthesis of *A. oryzae*. These results were in good agreement with morphological observations as well as gene expression profiling, which suggested that specific regulators were required for the transition from the adaptive phase to logarithmic and stationary phases. In addition, different developmental pathways converged to orchestrate the development of *A. oryzae*.

**Table 2:** The top 5 pathways with the highest rich factor

Pathway	GEGs	All genes	Qvalue	Pathway ID
Ao_24-vs-Ao_48				
Degradation of aromatic compounds	20	50	0.00012	ko01220
Tyrosine metabolism	24	71	0.000186	ko00350
Starch and sucrose metabolism	24	87	0.005467	ko00500
Steroid biosynthesis	14	41	0.008188	ko00100
Phenylalanine metabolism	15	46	0.008188	ko00360
Ao_24-vs-Ao_72				
Tyrosine metabolism	24	71	0.001257	ko00350
Pentose and glucuronate interconversions	19	51	0.001257	ko00040
Starch and sucrose metabolism	26	87	0.002514	ko00500
Glycolysis / Gluconeogenesis	21	65	0.002746	ko00010
Degradation of aromatic compounds	17	50	0.005013	ko01220
Ao_48-vs-Ao_72				
Carotenoid biosynthesis	2	5	0.033051	ko00906
Terpenoid backbone biosynthesis	2	27	0.423734	ko00900
Sesquiterpenoid and triterpenoid biosynthesis	1	5	0.423734	ko00909
Vitamin B6 metabolism	1	8	0.423734	ko00750
Valine, leucine and isoleucine degradation	2	45	0.423734	ko00280

### Clustering of gene expression profiles across the three growth stages

**Fig. 5:**
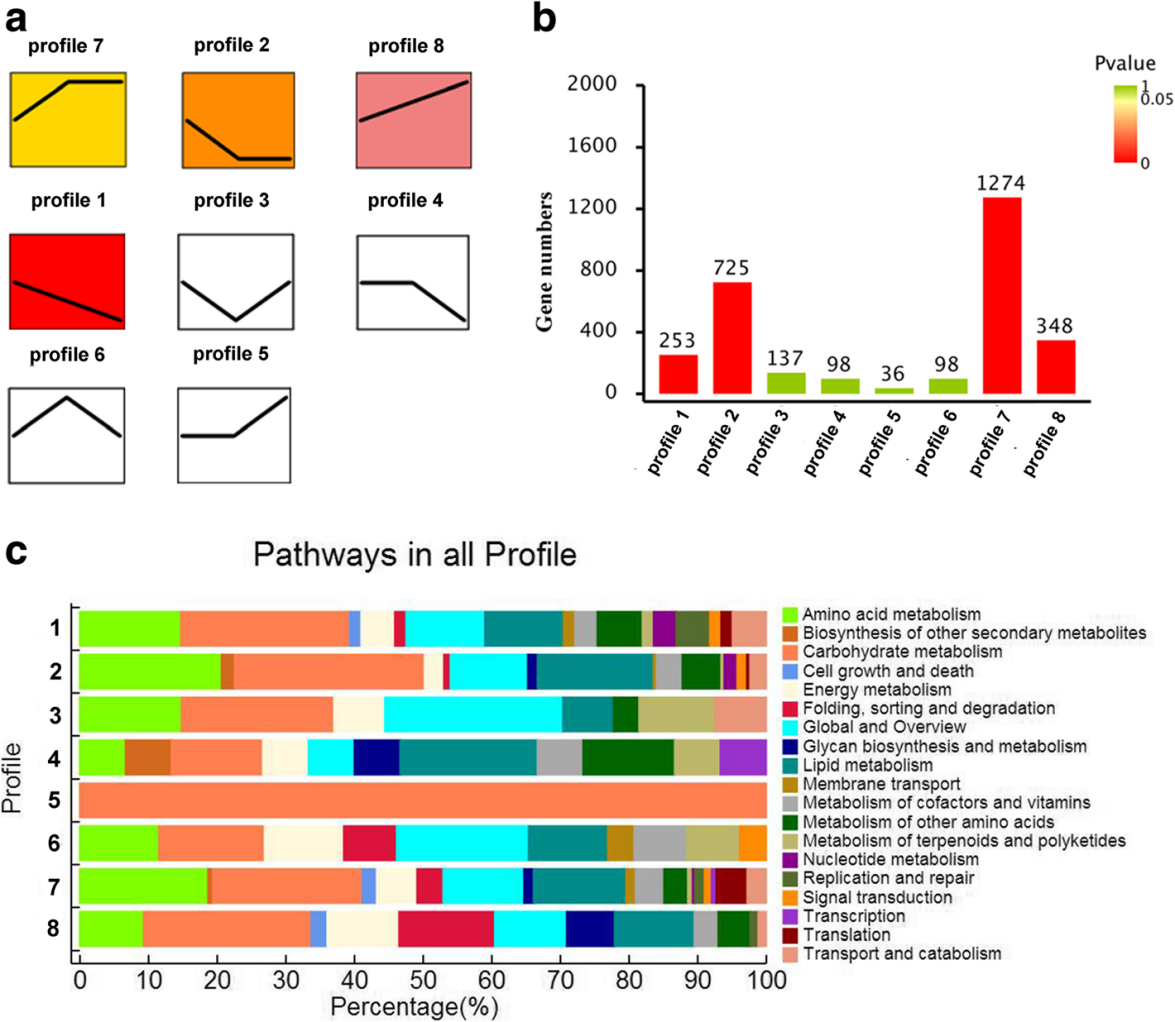
Clustering of gene expression profiles across three growth stages. **a** Eight gene clusters with different expression patterns. **b** The number of genes for each gene cluster. **c** Functional annotation of each gene clusters

The expression pattern of DEGs was further investigated through K-Means clustering of gene expression levels. A total of eight gene clusters with distinct expression patterns were identified (Fig. 5a). The results suggested that cluster 8 and 1 contained 348 and 253 transcripts respectively, which showed opposite expression patterns (Fig. 5a and b). Cluster 8 showed a gradual increase upon transition from the adaptive phase to the logarithmic and stationary phases, while cluster 1 showed a gradual decrease, indicating different roles with respect to the development of *A. oryzae*. Based on the functional annotation of the gene clusters, cluster 8 mainly belonged to carbohydrate metabolism, folding, sorting and degradation, while cluster 1 was mainly related to carbohydrate metabolism and lipid metabolism (Fig. 5c). Though cluster 8 and cluster 1 were both mainly related to carbohydrate metabolism, when using lower GO levels, cluster 8 mainly involved in galactose metabolism while cluster 1 mainly involved in glycolysis/gluconeogenesis. Cluster 7 had the most number genes (1274), mainly involved in carbohydrate and amino acid metabolism, which showed an increase from the adaptive phase to the logarithmic phase, and a steady trend from the logarithmic phase to the stationary phase. Furthermore, almost all genes of cluster 5 were involved in the carbohydrate metabolism, which was steady from the adaptive phase to the logarithmic phase, and showed an increase from the logarithmic phase to the stationary phase. These results were in good agreement with differential genes expression analysis, which suggested that specific gene expression and metabolism pathways were required for the transition from the adaptive phase to the logarithmic and stationary phases, while the developmental pathways were relatively steady during the transition from the logarithmic phase to the stationary phase. Additionally, the expression pattern of genes involving in the biosynthesis of polyketides, terpenoids and other secondary metabolites (mainly including indole diterpene alkaloid) were presented in the Fig. 5c. It is observed that genes involved in the metabolism of terpenoids and polyketides were mainly enriched in cluster 6, which showed an increase from the adaptive phase to the logarithmic phase, and a decrease trend from the logarithmic phase to the stationary phase. Genes involved in the metabolism of other secondary metabolites were mainly enriched in cluster 4, which showed a steady trend from the adaptive phase to the logarithmic phase, and a decrease trend from the logarithmic phase to the stationary phase.

## Discussion

Many studies have focused on the expression and regulation of developmental genes in fungi that might participate in the cell growth processes and morphogenesis, which have a significant influence on metabolic pathways and accumulation of metabolic products. The next generation sequencing technologies facilitates to study the regulatory mechanisms of developmental events in *A. oryzae*. Here, RNA-seq was applied to study the transcriptome of *A. oryzae*, which displayed the dynamic changes of distinct classes of genes during *A. oryzae* growth. By illustrating gene expression profiles at different growth stages, we provide a basis for studying the developmental processes in *A. oryzae*.

The morphology of *A. oryzae* is a subject of considerable interest, mainly due to the influence of morphology on process productivity. The morphological forms are generally described on at macroscopic and microscopic levels, where macromorphology describes the gross morphology, such as mycelia or spores, and the micromorphology describes the properties of the macromorphological forms [16, 17]. Kitamoto et al. described the detailed findings of cell biology of *A. oryzae* across developmental stagees at the microscopic level, and revealed that the microscopic morphology has a direct effect on metabolic pathway activity via co-regulation of genes, and influences productivity due to segregation of hyphae [3]. In this study, we described the changes in morphology and reported dynamic changes of distinct classes of genes and metabolic pathways during growth. This information would be helpful in improving our understanding of the possible biological processes and be beneficial to the industrial breeding. When the metabolic pathway activities during growth are better understood, strains with better productivity and physical properties of the culture can be engineered.

Previous studies have also focused on the relationship between the morphology and enzyme of *Aspergillus* fungi [7, 18, 19]. Wongwicharn et al. found that the concentration of oxygen increased during continuous cultivation of *A. niger* while investigating the heterologous enzyme secretion [20]. However, as metabolism, physiology and morphology are likely to be affected by the change in O_2_ levels, the resultant changes in production may not be due to the changes in morphology alone but both of physiology and morphology. We employed RNA-seq to study the transcriptome of *A. oryzae* upon changes in morphology of *A. oryzae* (Fig. 1) to better understand the dynamic changes of distinct classes of genes and metabolic pathways. We found that multiple pathways were altered in *A. oryzae* across the growth stages. From the adaptive phase to logarithmic phase, along with the color change from white to kelly green, the pathway of degradation of aromatic compounds were enriched among the highest DEGs. These genes were involved in degrading diverse chemical substances, including man-made chemicals in the environment, which are mostly aromatic compounds [21]. Phale et al. have reported that microbes have evolved the ability to utilize highly reduced and recalcitrant aromatic compounds as a potential source of carbon and energy [22]. Coincidentally, an aromatic hydrocarbon utilizing fungus, which was identified to the species level as *A. oryzae*, was isolated from a wastewater polluted sea spot in the Mediterranean, Alexandria, Egypt [23]. The highest proportion of DEGs from the logarithmic phase to the stationary phase was enriched in the carotenoid biosynthesis, terpenoid backbone biosynthesis as well as sesquiterpenoid and triterpenoid biosynthesis (Table 2). These results suggested that genes essential for growth and adaption to the environment were included at adaptive phase and logarithmic phase, while genes involved in secondary metabolites biosynthesis pathway were up-regulated during the stationary phase; the latter may also contribute to the transformation of the color of the organism to olive green.

In conclusion, our data illustrated the expression patterns of distinct classes of genes and characterized the functional characteristics of DEG sets between different developmental transitions of *A. oryzae*. The analyses captured the molecular signatures during *A. oryzae* growth, which will enhance the understanding of the developmental dynamics and provide guidance for the genetic improvement and industrial breeding.

## Methods

### Sample preparation and growth curve measure

Some enzymes, including heterologous proteins, can be produced at much higher levels by solid-substrate culture as compared to submerged liquid culture [24]. In addition, *A. orzyae* is often used as the solid-substrate fermentation. Therefore, the potato dextrose agar (PDA) plates were served as growth medium in our studies. Wild-type *A. oryzae* 3.042 (CICC 40092) was inoculated on fresh PDA medium in batch mode at 29 ± 1 °C for three days. Spores were then harvested and suspended in sterile water with 0.05% Tween-80, and the density of spores was counted using a hemocytometer. Freshly prepared suspension with 1 × 10^7^ conidia was inoculated on individual plates covered by cellophane (Solarbio, Beijing, China), and all cultures were placed at 30 °C in the dark. To assess the fungal viability, the plates were sampled every 12 h starting from the first 24 h after incubation to 72 h. Fungal mycelia were collected, and the biomass was measured after drying overnight; meanwhile, the spores were gathered, and their the density was determined by a hemocytometer. Based on the biomass and spore density, samples at 24, 48 and 72 h, corresponding to the adaptive phase, logarithmic phase and stationary phase of *A. oryzae*, were harvested by centrifugation (14,000 rpm, 4 °C, 1 min.) and immediately frozen in liquid nitrogen for RNA isolation. Three replicates were processed at each time point in the above experiments.

### RNA isolation, library construction and sequencing

After scraping the cell samples at different growth stages on the cellophane membrane, the total RNA was extracted with a fungal RNA kit (Omega Bio-tek, Norcross, GA, USA) coupled with DNA digestion, and mRNA was enriched by Oligo(dT) beads. The enriched mRNA was then fragmented into short fragments and reverse transcripted into cDNA using random primers. The cDNA fragments were purified using QiaQuick PCR extraction kit and ligated to Illumina sequencing adapters. The ligation products were size selected by agarose gel electrophoresis, amplified and sequenced using Illumina HiSeqTM2500. The transcriptome datasets are raw reads containing adapters or low quality bases. Therefore, to get clean reads, reads will be further filtered by removing reads containing adapters and more than 10% of unknown nucleotides (N). Low quality reads that contained more than 50% of low quality (Q-value≤20) bases were also removed. Furthermore, reads that mapped to ribosome RNA (rRNA) database using Bowtie2 were removed to get the final clean reads, which were further used for assembly and transcriptome analysis.

### Alignment with reference genome and differentially expressed genes analysis

The genome sequences (Accession number:AKHY00000000) and annotation files of *A. oryzae* 3042 were downloaded from NCBI [25]. Clean reads from each sample were mapped to the reference genome using TopHat2 (version 2.0.3.12) [26]. The reconstruction of transcripts was carried out with software Cufflinks, which together with TopHat2. To identify the new gene transcripts, all of the reconstructed transcripts were aligned to reference genome. The transcripts unknown or in the intergenic spacer region were defined as novel genes. The relative abundances of transcripts were quantified by software RSEM [27].

Gene expression levels were normalized using the FPKM (Fragments Per Kilobase of transcriptper Million mapped reads) method. To identify differentially expressed genes (DEGs) across samples, the edgeR package was used. We identified genes with a fold change ≥2 and a false discovery rate (FDR) < 0.05 in a comparison as significant differentially expressed genes [28]. DEGs were then subjected to enrichment analysis of GO functions and KEGG pathways.

### Alternative splicing analysis

The results of TopHat included all alternative splicing information and the junction structure was determined as follows: First, alternative splicing sites with less than 5 reads were filtered out. Then, the remaining alternative splicing sites were mapped to known alternative splicing sites (1 bp error was allowed) to identify known alternative splicing sites. Finally, the unmapped new alternative splicing sites were classified into 8 categories: Intergenic (junctions start from and/or end up with the area between genes), P5_splice (junctions start inside an exon, end up with initiation site of another exon), P3_splice (junctions start from an exon termination site and end up inside another exon), ES (junctions start from an exon termination site and end at another termination site), IR (junctions start and end in the same exon), AFS/TSS (junctions start from the 5′ end of exon initiation site and end up with another exon), ALS/TTS (junctions start at the 3′ end of the last exon initiation site and end up with another exon) and Others (junctions that do not belong to any of the above categories).

### Trend analysis

Gene expression pattern analysis is used to cluster genes of similar expression patterns for multiple samples (at least 3 in a specific time point, space, or treatment dose size order). To examine the expression pattern of DEGs, the expression data of each sample (in the order of treatment) were normalized to 0, log2(v1/v0), log2(v2/v0), and then clustered by Short Time-series Expression Miner software (STEM) [29]. The parameters were set as follows:

1) Maximum Unit Change in Model Profiles between Time Points is 1;

2) Maximum output profiles number is 20 (similar profiles will be merged);

3) Minimum ratio of fold change of DEGs is no less than 2.

The clustered profiles with *p*-value ≤0.05 were considered as significant profiles. Then the DEGs in all or each profile were subjected to Gene Ontology (GO) and KEGG pathway enrichment analysis. Through hypothesis testing based on p-value calculation and FDR correction, the GO terms or Pathways with Q-value ≤0.05were defined as significantly enriched GO terms or pathways.

### Functional annotation of DEGs

Gene Ontology (GO) is the framework for the model of biology and defines concepts used to describe gene function, and relationships between these concepts. The genes were compared against the NCBI non-redundant protein (NR) databases to predict and classify functions using the BLASTX program with an E-value cutoff at 1e-5, which described in the previous studies. The BLAST results were imported into Blast2GO software to retrieve associated GO terms [30]. We performed GO enrichment analysis to shortlist all GO terms that were significantly enriched in the DEGs when compared to the genome background, and DEGs that correspond to biological functions were filtered. GO terms were then assigned to the DEGs to produce an overview of the groups of genes present in the transcriptome for biological processes, molecular functions, and cellular components.

Genes usually interact with each other to participate in certain biological functions. Pathway-based analysis helps to further understand genes biological functions. KEGG is the major public pathway-related database. Genes were submitted to the KEGG Automatic Annotation Server (KAAS), and the single-directional best hit information method was selected. In this way, we identified significantly enriched metabolic pathways or signal transduction pathways in the DEGs when compared with the whole genome background using pathway enrichment analysis [31].

### Quantitative real-time PCR analysis

The expression of eight genes including *D9D1, D9D2, D9D3, D9D4, D12D1* and *D12D2*, which are involved in the linoleic acid biosynthesis in *A. oryzae*, was validated by real-time RT-PCR analysis. Real-time RT-PCR was performed using Real-Time PCR System (Bio-Rad). *GAPDH* served as the reference gene for normalization of the target gene expression and to correct for variation between samples. The PCR conditions were as follows: 95 °C for 2 mins, followed by 40 cycles of 95 °C for 10 s, 60 °C for 15 s and 72 °C for 20 s. Melting curve analyses of the amplification products were performed at the end of each PCR reaction to ensure that only specific products were amplified. Primers used for the candidate genes are listed in Additional file 6: Table S1. The comparative 2^−ΔΔCT^ method was employed to calculate relative expression between samples [32].

## Conclusion

In conclusion, the physiological and morphological differentiation of *A. oryzae* over the entire course of growth was characterized. Furthermore, RNA-seq was employed to better understand the transcriptional processes that occur during the growth stages. Most differential genes were expressed during the transition from the adaptive phase to logarithmic and stationary phases, while a relatively steady trend was obsreved for the transition from the logarithmic phase to stationary phase. A majority of these differentially expressed genes were enriched for single-organism process, metabolic process and catalytic activity. Additionally, genes essential for growth and adaption to the environment were enriched during the adaptive and logarithmic phases, while genes involved in secondary metabolite biosynthesis pathway were up-regulated during the stationary phase, which may contribute to the transformation to olive green phenotype. These results build a foundation for identifying developmentally important genes and for understanding the possible biological processes across various growth stages.

## Additional files

Additional file 1: **Figure S1**. The Pearson’s correlation coefficient of gene expression between repeats of each sample. Ao_24_1, 2, 3: samples at 24 h after incubation; Ao_48_1, 2, 3: samples at 48 h after incubation; Ao_72_1, 2, 3: samples at 72 h after incubation. (PNG 305 kb)

Additional file 2: **Figure S2**. Correlation analysis of qRT-PCR and transcriptome results. (TIFF 51 kb)

Additional file 3: **Table S2**. The expression levels and functional annotation of DEGs between Ao_24 and Ao_48. (XLS 1701 kb)

Additional file 4: **Table S3**. The expression levels and functional annotation of DEGs between Ao_24 and Ao_72. (XLS 1836 kb)

Additional file 5: **Table S4**. The expression levels and functional annotation of DEGs between Ao_48 and Ao_72. (XLS 215 kb)

Additional file 6: **Table S1**. qRT-PCR primers used in this study. (DOCX 16 kb)

## Abbreviations

A.oryzae: Aspergillus oryzae
DEGs: Differentially expressed genes
FDA: False discovery rate
FDA: Food and Drug Administration
FPKM: Fragments Per Kilobase of transcriptper Million mapped reads
GO: Gene Ontology
GRAS: Generally Recognized as Safe
KEGG: Kyoto Encyclopedia of Genes and Genomes
PDA: Potato dextrose agar
*S.cerevisiae*: *Saccharomyces cerevisiae*
WHO: World Health Organization
