# Supplementary figures and images for: Transcriptome analysis of different growth stages of Aspergillus oryzae reveals dynamic changes of distinct classes of genes during growth
Source: BMC Microbiol. 2018 Feb 14;18:12. doi: 10.1186/s12866-018-1158-z (PMC5813417; doi:10.1186/s12866-018-1158-z)

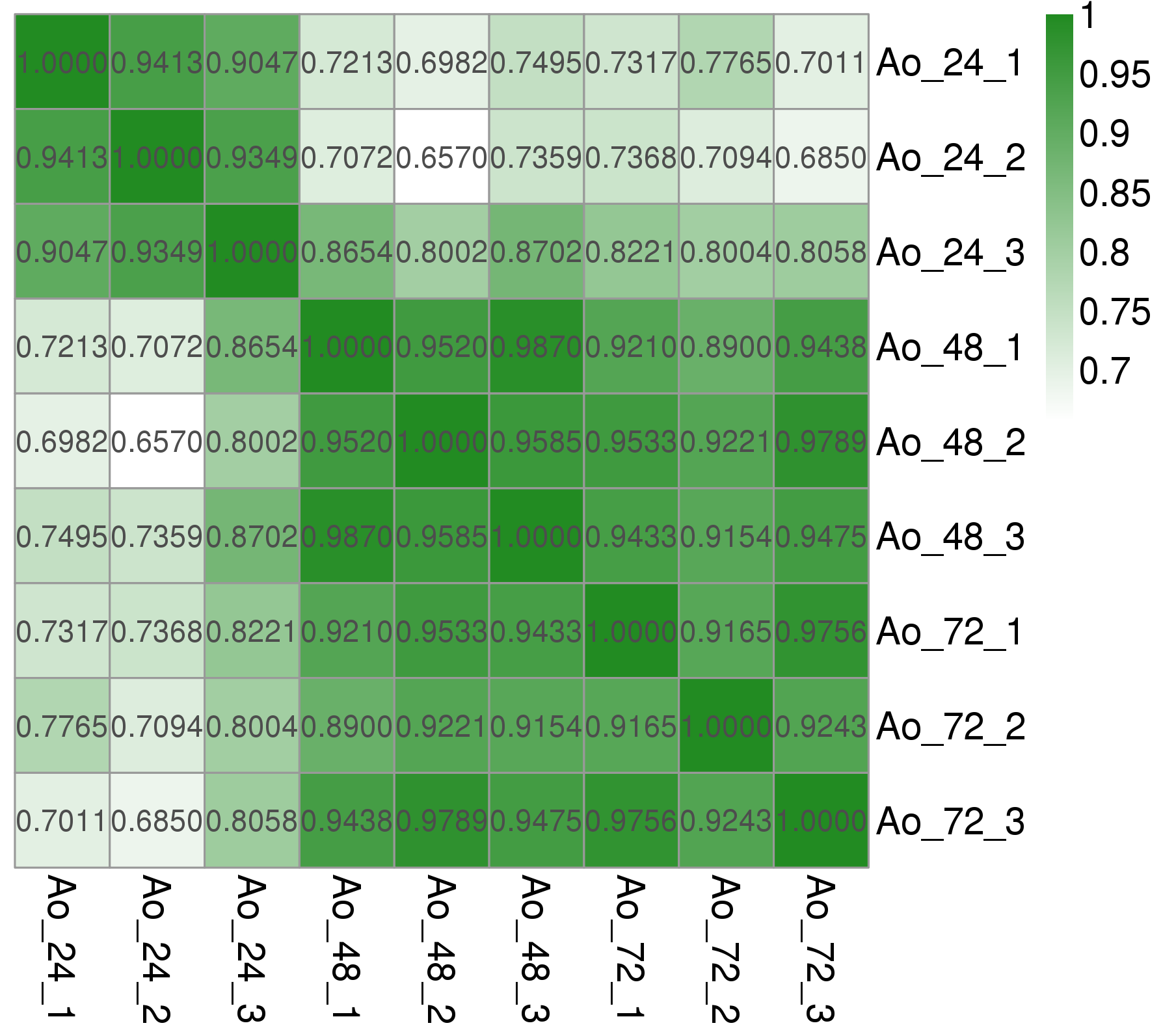

Supplement: Supplementary file 1 — Figure S1. The Pearson’s correlation coefficient of gene expression between repeats of each sample. Ao_24_1, 2, 3: samples at 24 h after incubation; Ao_48_1, 2, 3: samples at 48 h after incubation; Ao_72_1, 2, 3: samples at 72 h after incubation. (PNG 305 kb) [file 12866_2018_1158_MOESM1_ESM.png]

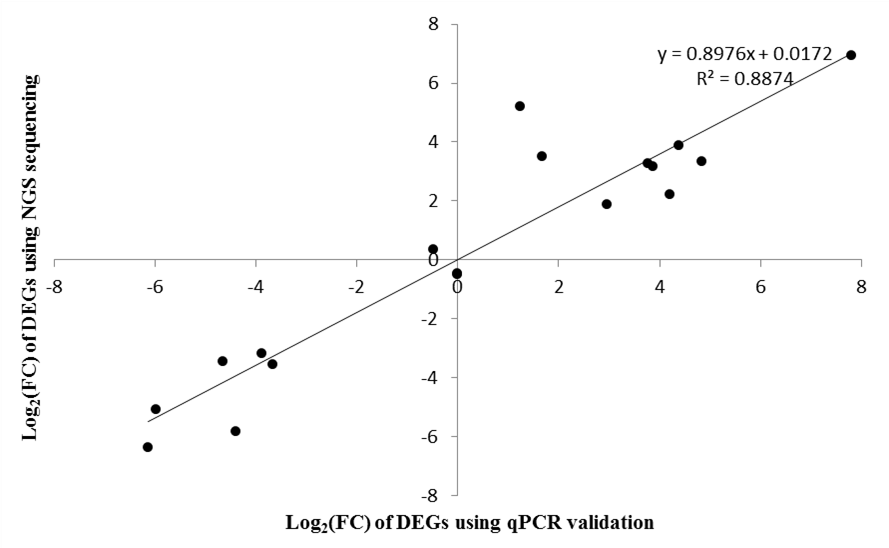

Supplement: Supplementary file 2 — Figure S2. Correlation analysis of qRT-PCR and transcriptome results. (TIFF 51 kb) [file 12866_2018_1158_MOESM2_ESM.tif]
