# Supplementary material for: Transcriptome analysis of different growth stages of Aspergillus oryzae reveals dynamic changes of distinct classes of genes during growth
Source: BMC Microbiol. 2018 Feb 14;18:12. doi: 10.1186/s12866-018-1158-z (PMC5813417; doi:10.1186/s12866-018-1158-z)
Supplement: Supplementary file 6 — Table S1. qRT-PCR primers used in this study. (DOCX 16 kb) [file 12866_2018_1158_MOESM6_ESM.docx]

**Table S1. qRT-PCR primers used in this study**

| **Primer** | **Sequence (5′-3′)** | **Purpose** |
| --- | --- | --- |
| D9D1-F | AGCAGAACCCCAAGCGTATT | qRT-PCR of D9D1 |
| D9D1-R | CAGAAAGTTGCCTGCTGGAC | qRT-PCR of D9D1 |
| D9D2-F | CGTCATAACCGCCTTCGTAA | qRT-PCR of D9D2 |
| D9D2-R | TGTTGGATGCGACCTTTTTC | qRT-PCR of D9D2 |
| D9D3-F | CCCTGGTCCTGTCAGTCGTA | qRT-PCR of D9D3 |
| D9D3-R | CACGCTGTATGGGTCAAGGT | qRT-PCR of D9D3 |
| D9D4-F | CGACCAGCCCTACGATGATA | qRT-PCR of D9D4 |
| D9D4-R | GTCTGGGGCCTTCTTCAAAC | qRT-PCR of D9D4 |
| D12D1-F | GGGATTCTTCAAGGCTCTCTTC | qRT-PCR of D12D1 |
| D12D1-R | CTCCGATGCCGTTGGTATTA | qRT-PCR of D12D1 |
| D12D2-F | CTGATCGGTCACCAGCTATTC | qRT-PCR of D12D2 |
| D12D2-R | TTGCAGGTCCCATCTTTCC | qRT-PCR of D12D2 |
